# Back-transformations in random-effects meta-analysis—Impact and interpretation

**DOI:** 10.1017/rsm.2026.10096

**Published:** 2026-05-15

**Authors:** Jan-Bernd Igelmann, Markus Pauly, Wolfgang Viechtbauer

**Affiliations:** 1 Department of Statistics, TU Dortmund University, Germany; 2 Research Center Trustworthy Data Science and Security, UA Ruhr, Germany; 3 Department of Psychiatry and Neuropsychology, Maastricht University, Netherlands

**Keywords:** back-transformation, integral transformation, meta-analysis, random-effects model, transformation bias

## Abstract

In meta-analyses, effect size measures with bounded or non-normal sampling distributions are commonly analyzed on a transformed scale to justify normality assumptions. While point estimates and confidence intervals (CIs) are routinely back-transformed to the original scale for interpretation, this practice is nontrivial in random-effects models. In particular, standard inverse back-transformations yield estimates of the median rather than the mean effect size due to Jensen’s inequality. Integral back-transformations provide a principled solution for recovering the mean on the original scale, but their use entails practical issues. We study integral back-transformations for several effect size measures, including correlation coefficients, proportions, odds and risk ratios, and Cronbach’s alpha. We derive general formulations for integral back-transformations and corresponding CIs that are applicable across different transformation functions and provide a software implementation. Although required to obtain correct mean estimates, these approaches must be used with caution, as they are sensitive to heterogeneity estimation and can be unstable for unbounded transformations. Certain asymmetric transformations can also lead to inconsistent inference results. We illustrate these issues using analytical considerations and re-analyses of several meta-analyses. Importantly, we stress that the choice of back-transformation depends on the analyst’s goals. The standard inverse back-transformation remains well suited for descriptive purposes and is often preferable in practice, provided that it is correctly interpreted as a back-transformed median effect size. We recommend using the integral back-transformation only when a mean estimate is explicitly required, and restricting its use for CIs to cases where they are demanded, but not for hypothesis testing.

## Highlights

### What is already known?

- In random-effects meta-analysis, standard inverse back-transformations do not recover the mean effect size on the original scale but correspond to the median with the resulting transformation bias increasing with heterogeneity.
- Integral back-transformations provide a principled way to obtain the mean effect size on the original scale.

### What is new?

- We introduce a general formulation of the integral back-transformation for estimating the mean effect size and a corresponding confidence interval.
- We emphasize that these methods must be used with caution and should only be applied when the estimand is explicitly the back-transformed mean effect size.
- We clarify that standard inverse back-transformations often remain preferable, especially for descriptive purposes, when they are correctly interpreted and reported as back-transformed median effect sizes.
- We illustrate and quantify the differences between standard and integral back-transformations using several meta-analytic examples.

### Potential impact for RSM readers

- The article contains a general methodological discussion of back-transformations in random-effects meta-analysis. An application is possible in all areas of meta-analysis.
- Commonly used transformations for different effect sizes, which are used in various research areas, are presented.
- Implementations of the back-transformations and the data examples are available in R packages metafor and metadat, respectively.

## 1 Introduction

Broadly speaking, a meta-analysis consists of quantifying the evidence about some phenomenon of interest (e.g., the size of the difference between a treatment and a comparison group, and the strength of the association between two variables) in terms of an effect size measure (e.g., a risk or odds ratio, and a correlation coefficient) for a set of relevant studies and then summarizing the collection of estimates using appropriate statistical models.

In practice, meta-analyses are applied to various effect size measures, where a normal distribution is typically assumed for the sampling distributions of the individual estimates.^1^^–^ ^3^ However, some effect sizes, including, for example, correlation coefficients or those derived from binary data, have a bounded distribution with a variance potentially influenced by their own magnitude. Thus, a normal sampling distribution can hardly be justified for such measures, especially when the sample sizes of the individual studies are not large.^3^ A commonly used solution is to apply a transformation to the estimates before the analysis, that is, the effect sizes are transformed to a real-valued scale using a continuous, monotonically increasing, and non-linear function. Popular transformations are, for example, *Fisher’s r-to-z* transformation for correlation coefficients (see Borenstein et al., Chapter 6)^1^ or the *log* transformation for risk and odds ratios obtained from 2×2 tables (see Borenstein et al., Chapter 5).^1^ Subsequently, all estimations are performed based on the transformed values.

A crucial part of this two-step procedure is the back-transformation, which is nontrivial in a random-effects model, or equivalently, when heterogeneity is modeled. Estimates or confidence intervals (CIs) are not easily interpretable on the transformed scale. The standard procedure is to apply the inverse transformation function to the various quantities of interest (e.g., the mean effect size estimate and the bounds of the corresponding CI). With this article, we want to highlight that such non-linear back-transformations do not in general lead to estimates of the mean effect size on the original (untransformed) scale due to Jensen’s inequality. Instead, the back-transformation process results in an estimate of the *median effect size* of the back-transformed distribution.

This observation is not new. The handbook of Schmid et al. (see p. 78)^2^ notes that this back-transformation corresponds to the median. Jackson and White^3^ make a remark regarding the so-called “transformation bias.” However, many researchers dealing with meta-analyses either remain unaware of or overlook this fact. Therefore, erroneous statements are often made and biased estimates of the mean are reported. A detailed discussion in the context of the correlation coefficient in meta-analyses together with a methodological precise solution in the form of an integral back-transformation is given by Hafdahl.^4^ Strategies to back-transform the bounds of CIs are given as well. This integral back-transformation was included in simulation studies by Welz et al.^5^ and also Hafdahl.^6^

In the present work, we draw attention to methods for the correct back-transformation of the mean for various effect size measures. We also suggest a correct integral back-transformation for transformations that do not map to the whole real scale, such as the variance stabilizing *arcsine* transformation of proportions based on Anscombe.^7^

Such integral transformations are required to obtain correct estimates of the mean or other higher-order moments. Despite this, we caution analysts against using integral transformations blindly. They are sensitive to uncertainty in the estimation of the degree of heterogeneity and can be highly unstable for unbounded transformations, such as the *log* transformation. They should only be used when an estimate of the mean is explicitly required by the reporting strategy or when subsequent analyses based on this mean as estimand are performed, that is, additive extrapolations. Furthermore, we discuss an alarming lack of invariance that can be harmful in interpreting results correctly when the transformation lacks point symmetry around a reference value. This also affects statistical inference for the back-transformed mean. We explicitly emphasize that hypothesis tests and CIs should be constructed on the transformed scale, except in cases where a CI for the back-transformed mean effect size is explicitly required.

In this context, we analyze several examples that clarify the risks associated with applying the integral back-transformation. The corresponding data sets contain different outcome structures (proportions, Pearson correlation coefficients, binary 2×2 data, and Cronbach’s α values), and thus different transformation functions are presented. Furthermore, we quantitatively analyze the differences between the two back-transformations and point to a software implementation of the integral back-transformations for all presented transformations.

## 2 Methods

In this article, we focus on the standard (“normal–normal”) random-effects model for meta-analysis which accounts for between-study heterogeneity by means of a random effect. For the common-effect model, the differences between back-transformations are not present. Consider i=1,…,k available studies with $y_{i}$ denoting the effect size estimate in the *i*th study. We assume that some kind of transformation function (e.g., *log*) was applied to obtain the $y_{i}$ values. The transformation function is denoted by g:D→V . Here, D corresponds to the possible values of the measured effect size before transformation, and V⊆R is often equal to R but can also be a subset for certain transformations.

The random-effects model is given by

(2.1) $$\begin{array}{r} y_{i}=\mu +u_{i}+\varepsilon_{i}, \end{array}$$

where $u_{i}∼N(0,\tau^{2})$ and $\varepsilon_{i}∼N(0,v_{i})$ . The study-specific *within-study* variances $v_{i}$ are assumed to be known. $\tau^{2}\geq 0$ is called the *between-study* variance, quantifying the amount of heterogeneity. Again, when no heterogeneity is modeled ( $\tau^{2}$ is set or estimated to be zero) no random effects are present and the differences between the back-transformation approaches do not exist. In the commonly applied two-step estimation procedure for random-effects models of this form (see Borenstein et al., Chapter 12),^1^ the first step involves estimating $\tau^{2}$ . Throughout this article, we use the restricted maximum-likelihood estimator,^8^ although this particular choice is not material to the discussion at hand. Let ${\hat{\tau }}^{2}$ denote the corresponding estimate. Subsequently, the mean effect size μ is estimated via weighted least squares as

(2.2) $$\begin{array}{r} \hat{\mu }=\frac{\sum_{i=1}^{k}w_{i}y_{i}}{\sum_{i=1}^{k}w_{i}} \end{array}$$

with weights $w_{i}=(v_{i}+{\hat{\tau }}^{2}{)}^{-1}$ . The CIs for μ presented within this article are Wald-type CIs with lower and upper bounds for an α -level CI defined as $\left({\hat{\mu }}^{\left(l\right)},{\hat{\mu }}^{\left(u\right)})=\hat{\mu }\pm q_{1-\alpha /2}\cdot \text{SE}[\hat{\mu }\right]$ , where $q_{1-\alpha /2}$ denotes the (1−α/2) -quantile of the standard normal distribution and $\text{SE}[\hat{\mu }]={\left(\sum_{i=1}^{k}w_{i}\right)}^{-1/2}$ . For the example meta-analyses presented later, we use α=0.05 and hence report 95% CIs.

### 2.1 Back-transformation

The random-effects model assumes that the true effect sizes $\theta_{i}$ are normally distributed with expected value μ and variance $\tau^{2}$ (i.e., $\theta_{i}∼N(\mu ,\tau^{2})$ ). However, the estimates $\hat{\mu }$ and ${\hat{\tau }}^{2}$ that characterize this distribution apply to the transformed scale. We consider a simple illustrative example in which a *log* transformation is applied to risk ratios (RR), which corresponds to the special case of the well-known log-normal model. An estimate of $\hat{\mu }=0.58$ implies that we estimate that the log risk ratio is, on average, equal to 0.58, or equivalently, that the log risk is, on average, 0.58 points higher in the first compared to the second group. Such a finding is difficult to interpret and hence it is common practice to back-transform especially the estimate of the mean effect. Due to Jensen’s inequality, $g^{-1}(E[\theta_{i}])\neq E[g^{-1}(\theta_{i})]$ and hence $g^{-1}(\hat{\mu })$ is *not* an estimate of the mean effect on the back-transformed scale. As a result, the value exp⁡(0.58)=1.79 cannot be interpreted as indicating that the risk is, on average, 1.79 times higher (or 79% larger) in the first group compared to the second group. The value is the median and not the mean of the asymmetric (here specifically log-normal) distribution of the true effects on the back-transformed scale.

However, the mean can be back-transformed via an integral transformation. This has been proposed for correlation coefficients in meta-analyses by Hafdahl^4^ and generally works as follows. Assume that V=R holds for our transformation g , then the appropriate back-transformation is given by

(2.3) $$\begin{array}{r} \psi_{g}(\mu ,\tau^{2})=\frac{1}{\tau }\int_{-\infty }^{\infty }g^{-1}(t)\;\;\phi \;\left((t-\mu )/\tau \right)\;dt, \end{array}$$

where ϕ(⋅) is the density of the standard normal distribution. Formally, this equation represents the expected value of $g^{-1}(T)$ with T following a normal distribution with parameters μ and $\tau^{2}$ . Therefore, $\psi_{g}(\hat{\mu },{\hat{\tau }}^{2})$ is an estimate of the mean of the distribution of $g^{-1}(\theta_{i})$ . For example, suppose ${\hat{\tau }}^{2}=0.041$ in the example above, then $\psi_{g}(\hat{\mu }=0.58,{\hat{\tau }}^{2}=0.041)=1.82$ would indicate that the risk is, on average, 1.82 times higher in the first group compared to the second group.

Note that this bias is not present for quantiles of the back-transformed distribution due to the monotonicity of $g^{-1}(\cdot )$ . Therefore, since $\hat{\mu }$ is not only an estimate of the mean but also the median of the distribution of $\theta_{i}$ , $g^{-1}(\hat{\mu })$ is an estimate of the *median* of the distribution of $g^{-1}(\theta_{i})$ . Thus, it would be correct to say that in 50% of the cases (studies), the true effect is estimated to be above 1.79 (and below in the other 50% of cases).

A corresponding CI for the mean effect size is given by the integral transformed bounds $\psi_{g}({\hat{\mu }}^{\left(l\right)},{\hat{\tau }}^{2})$ and $\psi_{g}({\hat{\mu }}^{\left(u\right)},{\hat{\tau }}^{2})$ . CIs for the median effect size can again be obtained by applying the inverse back-transformation to the bounds $g^{-1}({\hat{\mu }}^{\left(l\right)})$ and $g^{-1}({\hat{\mu }}^{\left(u\right)})$ due to the monotonicity. Ambiguities due to one-sided transformation bias for certain transformations that are not point-symmetric around a specific neutral value may arise when using the proposed CI for the back-transformed mean as a basis for hypothesis testing. These issues are discussed in detail and illustrated with an example in Sections 3 and 4. Therefore, we generally recommend performing testing on the transformed scale where the normal–normal model is originally assumed. For the CI based on the $g^{-1}$ back-transformation corresponding to the median, such problems do not occur. It can be considered equivalent to conducting a test of the median on the transformed scale.

### 2.2 Back-transformation for transformations with different ranges

There are also transformations with V⊊R . For example, the range of values transformed by the *arcsine* transformation is V=[0,π/2]⊊R . In these cases, the normality assumption on the transformed scale is questionable. An advantage of the *arcsine* transformation, and a reason for its continued use is its variance stabilizing property.^7^

Due to the restricted range, an integration as in Equation (2.3) is no longer meaningful to estimate the back-transformed mean effect. In practice, the probability that lies outside of the restricted range is ignored, and therefore a truncated normal distribution is obtained. Therefore, we propose the following integral transformation for general transformations g:D→V⊆R :

(2.4) $$\begin{array}{r} \psi_{g}(\mu ,\tau^{2})=\frac{1}{\tau }\int_{a}^{b}g^{-1}(t)\;\frac{\phi \;\left((t-\mu )/\tau \right)}{\Phi ((b-\mu )/\tau )-\Phi ((a-\mu )/\tau )}\;dt, \end{array}$$

where Φ denotes the cumulative distribution function of a standard normal distribution. The integration limits are defined as a=infV and b=supV . In the special case V=R , Equation (2.4) reduces to Equation (2.3).

This implies that the assumed normal distribution, which is not strictly accurate, is truncated and subsequently rescaled to the range specified by the transformation function. Therefore, in cases with V⊊R , we are not estimating $E[g^{-1}(\theta_{i})]$ with $\theta_{i}∼N(\mu ,\tau^{2})$ as implied by the model from Equation (2.1) but with $\theta_{i}∼\text{TN}(\mu ,\tau^{2};a,b)$ . Here, TN(⋅;a,b) denotes the truncated normal distribution on the interval [a,b] . In many cases, especially when dealing with skewed distributions, the implicitly assumed distribution on the back-transformed scale can exhibit complex and non-intuitive shapes. Moreover, truncation introduces an additional bias when unequal probability masses are ignored on the left- and right-hand side of the distribution. It should also be noted that, due to truncation, the back-transformation via $g^{-1}$ does not yield the median of the TN distribution.

### 2.3 Specific transformations and R support

A comprehensive overview of commonly used transformations can be found in Table 1. Both, the standard inverse and the integral back-transformation, are implemented for all of these transformations in the R package metafor.^9^ The software name of the integral back-transformation function is denoted within the table. The respective functions for the standard $g^{-1}(x)$ transformations have the same name without the appended .int. For some transformations, a closed form of the integral back-transformation exists. For example, the *log* transformation leads to $\psi_{\log}(\mu ,\tau^{2})=\exp\left(\mu +\tau^{2}/2\right)$ . Otherwise, the integral in Equations (2.3) or (2.4) can be calculated numerically.

**Table 1:** Overview of commonly used transformations and their back-transformations Table 1 Long description.

Transformation	g(x)	$g^{-1}(x)$	D→V	$\psi_{g}$ in metafor	Applications
log	log⁡(x)	exp⁡(x)	(0,∞)→R	transf.exp.int	Ratios (odds, risk, … )
logit	$\log\left(\frac{x}{1-x}\right)$	$\frac{1}{1+e^{-x}}$	(0,1)→R	transf.ilogit.int	Proportions/
probit	$\Phi^{-1}(x)$	Φ(x)	(0,1)→R	transf.iprobit.int	probabilities
arcsine	$\text{arcsin}(\sqrt{x})$	${\text{sin}}^{2}(x)$	[0,1]→[0,π/2]	transf.iarcsin.int	
Fisher’s *r*-to-*z*	artanh(x)	tanh(x)	(−1,1)→R	transf.ztor.int	Correlations
AHW	$1-(1-x{)}^{1/3}$	$1-(1-x{)}^{3}$	[0,1]→[0,1]	transf.iahw.int	Alpha coefficients
ABT	−log⁡(1−x)	1−exp⁡(−x)	[0,1)→[0,∞)	transf.iabt.int	

*Note*: is the Φ cumulative distribution function of the standard normal distribution.

Other listed transformations are the *logit*, *probit*, and *arcsine* transformations. These are particularly useful for proportions and probabilities. Furthermore, *Fisher’s r-to-z* transformation for correlations is included. Another effect size of interest is Cronbach’s α , a measure of internal consistency that indicates how well the items in a test or questionnaire reliably measure the same underlying construct. It is often a component of meta-analysis in psychology and requires special transformations due to its often skewed distribution, especially with small samples. We added two common transformations for Cronbach’s α , the first based on Hakstian and Whalen^10^ (AHW) and the second based on Bonett^11^ (ABT). Note that the versions of these two transformations differ slightly from their original formulations to ensure that g(x) remains a monotonically increasing function in α .^9^ Of the transformations reported, those with a bounded V are the *arcsine*, *AHW*, and *ABT* transformations.

## 3 Analysis of the difference between both back-transformations

As noted above, the interpretation of $g^{-1}(\hat{\mu })$ as the estimated mean effect size is incorrect. Compared with the correct back-transformation $\psi_{g}(\hat{\mu },{\hat{\tau }}^{2})$ , the resulting transformation difference can be quantified by $g^{-1}(\hat{\mu })-\psi_{g}(\hat{\mu },{\hat{\tau }}^{2})$ . This, sometimes called transformation bias,^3^ is influenced by both the degree of heterogeneity and the shape of the transformation function at the point $\hat{\mu }$ .

**Figure 1:**
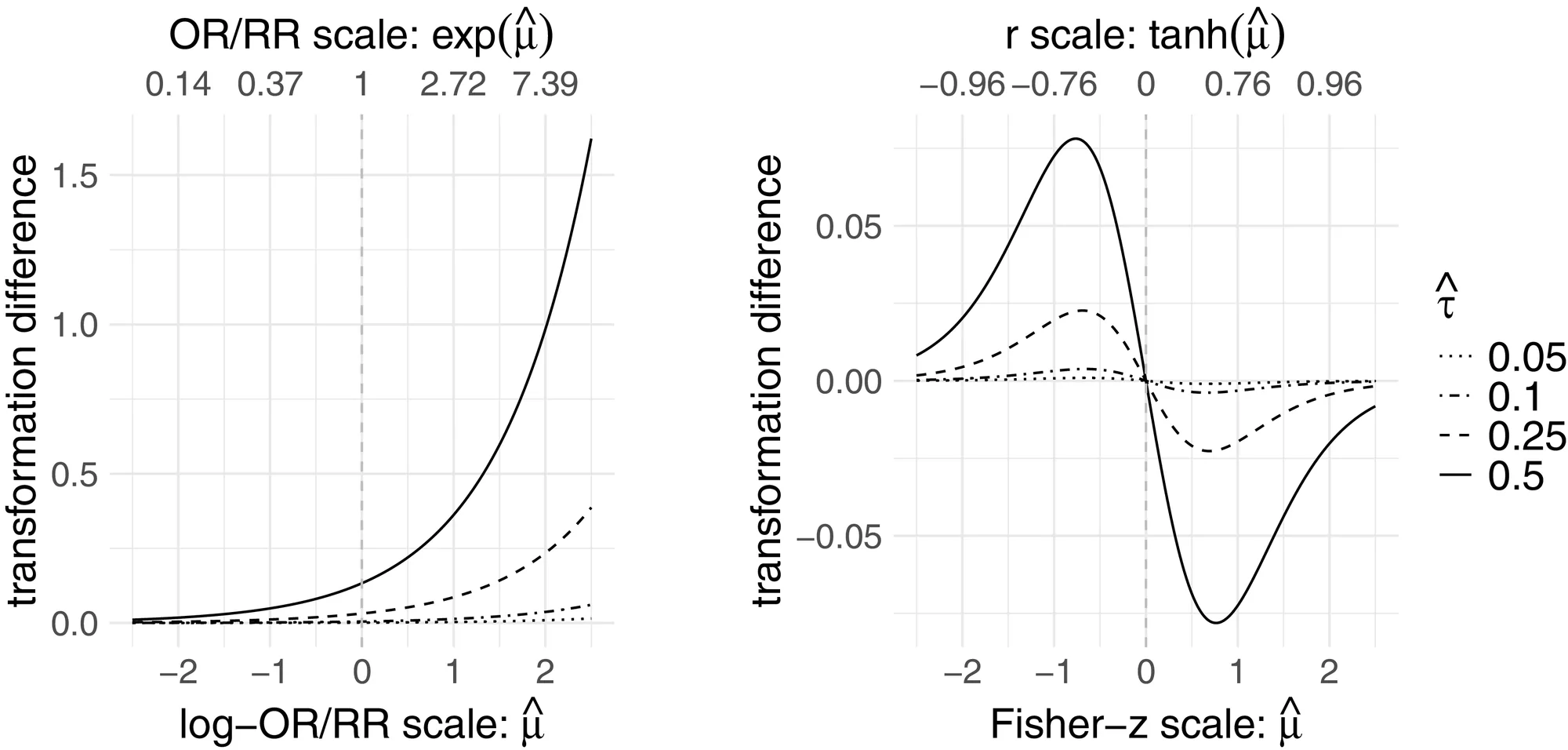
Visualization of the transformation difference $\psi_{g}(\hat{\mu },{\hat{\tau }}^{2})-g^{-1}(\hat{\mu })$ for the *log* transformation (left) and *r-to-z* transformation (right). Figure 1 Long description.

Figure 1 visualizes the difference for the *log* transformation (left plot) and *Fisher’s r-to-z* transformation (right plot) as a function of $\hat{\mu }$ (and $g^{-1}(\hat{\mu })$ ) for different values of $\hat{\tau }$ . In analyzing these plots, two properties require particular attention: *boundedness of the bias* and point symmetry around the origin. The *r-to-z* transformation is an example that exhibits both properties, whereas the *log* transformation does neither.

**Figure 2:**
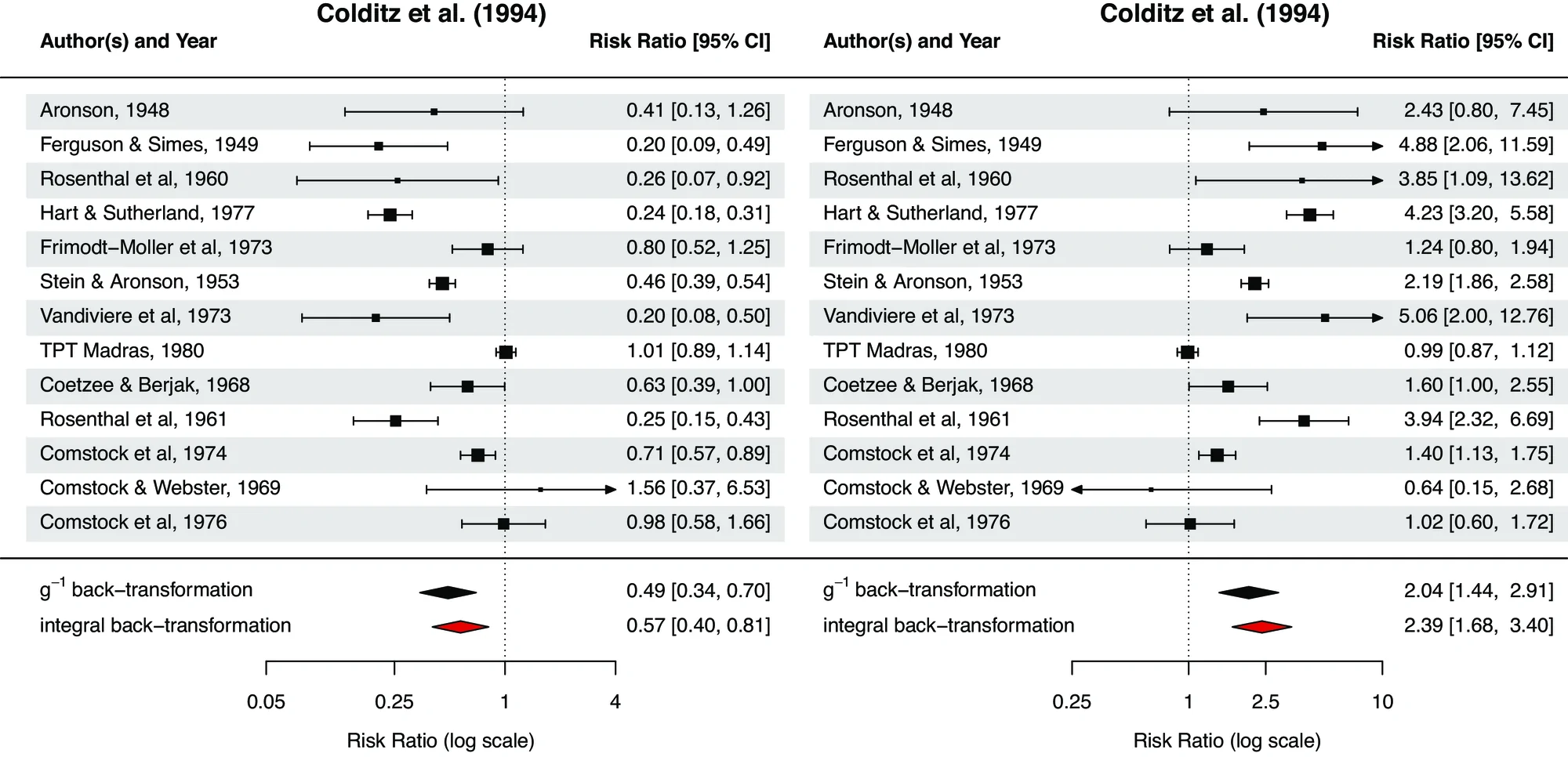
Forest plots for the Colditz et al. meta-analysis^15^ with the standard $g^{-1}$ and integral back-transformations; $\hat{\tau }=0.56$ ; definitions of the RR: vaccinated over control (left) and control over vaccinated (right). Figure 2 Long description.

**Figure 3:**
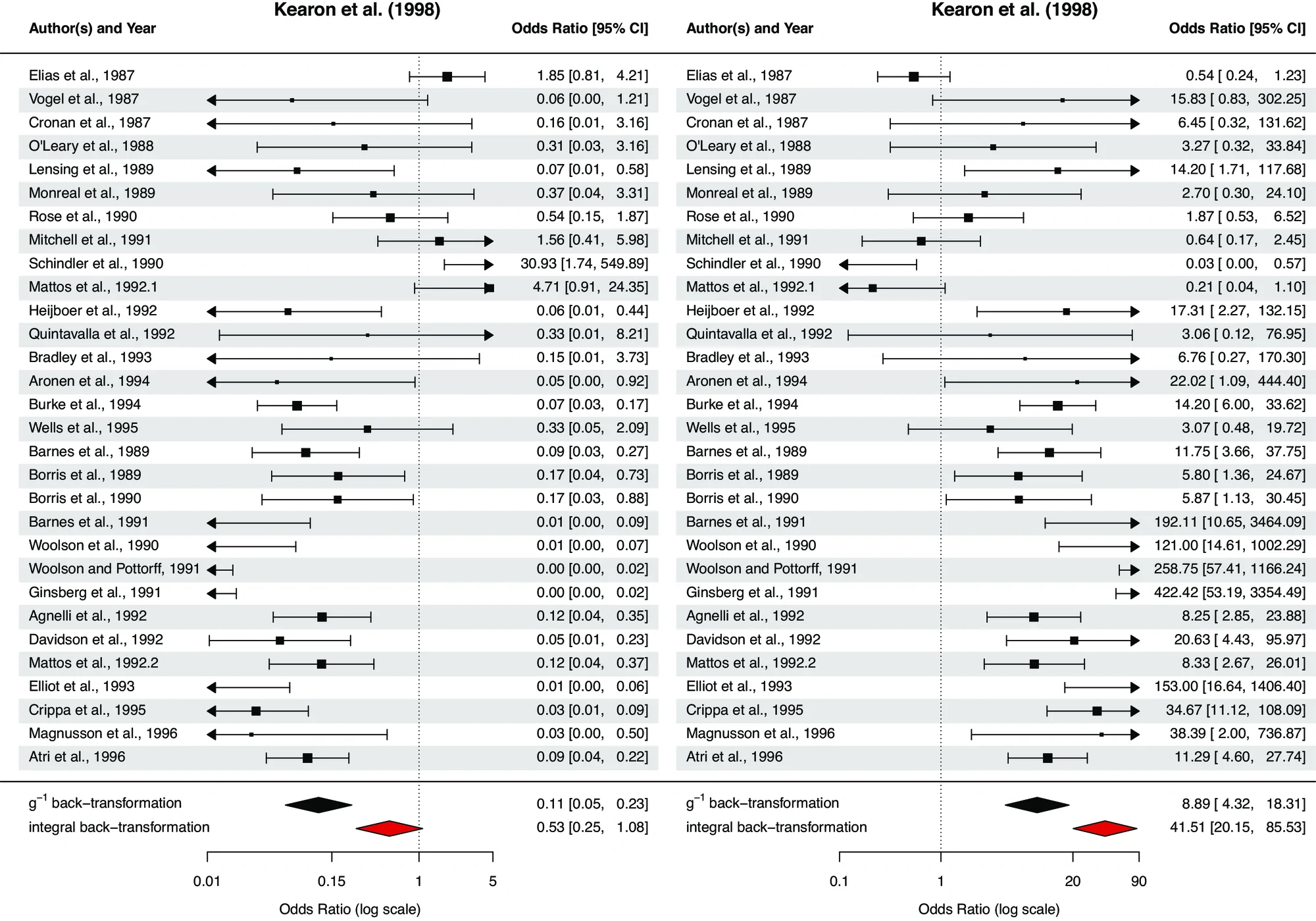
Forest plots for the Kearon et al. meta-analysis^16^ with the $g^{-1}$ and integral back-transformations; $\hat{\tau }=1.76$ ; definitions of the OR: positive over negative patients (left) and negative over positive patients (right). Figure 3 Long description.

For transformations defined over bounded *D*, the transformation bias is also bounded, and thus generally less extreme. For example, under the *r-to-z* transformation, the absolute value of the bias is limited for any fixed $\hat{\tau }$ (see the right panel in Figure 1). In contrast, the *log* transformation, which is defined over the positive real numbers, exhibits an exponentially increasing difference as $\hat{\mu }$ grows (see the left panel in Figure 1). For the *log* transformation, it is possible to present the integral back-transformation and based on this also the transformation difference in a closed form (see Section 2.3). The relative increase in transformation difference is equal to $\exp({\hat{\tau }}^{2}/2)$ and is therefore sensitive to the possibly imprecise estimation of τ , even more than the rest of the analysis. This represents a critical limitation of using the integral back-transformation in this setting. With such unbounded transformations, the integral back-transformation can become unstable and a careful decision for the choice of the back-transformation is needed. This is further illustrated in the two examples in the following section (see Section 4; Figures 2 and 3).

Additionally, the transformation difference of the *r-to-z* transformation is point-symmetric around the origin. This property is not shared by transformation functions that lack point symmetry around a neutral value depending on the effect size, namely, transformations for which symmetry around the midpoint of the effect-size domain does not hold. Formally, point symmetry around a midpoint c is defined by g(c+x)=−g(c−x) for all x∈[0,c−a) , where [a,b] denotes the domain and c=a+(b−a)/2 . This notion of point symmetry should not be confused with the symmetry defined for transformations of probabilities via g(p)+g(1−p)=const , which relates to invariance under exchanging events and non-events rather than exchanging groups.^12^

As a consequence, invariance of point estimates and inference can be violated due to the integral back-transformation. This can lead to problematic and counterintuitive results, that is, when inverting effect sizes. A particularly illustrative example involves the *log* transformation of odds ratios (ORs) when testing for the presence of a treatment effect. A commonly tested $H_{0}$ states that the mean OR is equal to one. Due to the skewness of the distribution on the exponentially back-transformed scale and the resulting systematic (rightward) transformation bias, the test outcome can depend on whether ORs are defined in one direction or its inverse. This issue is described in more detail in the applied example in Section 4.2.

Therefore, we do not recommend conducting hypothesis tests for the back-transformed mean and suggest constructing CIs only when they are explicitly required. This applies to transformations exhibiting the described asymmetry. Alternative approaches can be employed either by formally conducting inference for the mean on the transformed scale or by performing a standard median-based test using the inverse transformation $g^{-1}$ .

Equivalent plots of the transformation difference for all other transformations, which are listed in Table 1, are provided in Section A of the Supplementary Material. When comparing the transformation difference for the *logit* and *probit* transformation, it can be seen that the absolute difference of the *logit* transformation is larger across almost all combinations of $\hat{\mu }$ and $\hat{\tau }$ . The same applies for the difference of the *AHW* transformation compared with the difference of the *ABT* transformation. A direct comparison of the *arcsine* transformation with the *logit* and *probit* transformation is not meaningful due to the differing ranges of the transformations and the associated slight variation in the interpretation of $\psi_{g}$ .

## 4 Data application

To illustrate the impact of transformation difference and demonstrate the application of different types of transformations, we re-analyzed several data sets with different effect sizes. We begin by presenting two case studies applying the *log* transformation, which, as described in Section 3, can induce particularly extreme transformation difference and also conflicting test results for tests based on back-transformed CIs. The first data set (Section 4.1) exhibits a moderate, commonly observed level of heterogeneity, where a noticeable transformation difference is already evident. The second data set (Section 4.2) displays an extremely high level of heterogeneity, illustrating the issue of divergent test decisions, which has been discussed in Section 3. Additional examples including the usage of further effect sizes and corresponding transformations are summarized in Section 4.3. All analyzed data sets are available in the R package metadat.^13^ All analyses were conducted in R ^14^ (version 4.5.1) using the package metafor.^9^ The corresponding R script can be found in the Supplementary Material.

### 4.1 Detailed case study on *log* transformations for Colditz et al.

We illustrate the use of the *log* transformation with two data sets reporting 2×2 contingency data. One of these is the data set from Colditz et al.,^15^ which includes studies on the effectiveness of the Bacillus Calmette–Guérin vaccine against tuberculosis (TB). Reported are the numbers of TB-positive cases in the vaccinated and unvaccinated groups (“tpos” and “cpos,” respectively), as well as the numbers of TB-negative cases in both groups (“tneg” and “cneg”). The analysis is carried out using the *log* transformation applied to the RR, which are defined as: RR=(tpos/(tpos+tneg))/(cpos/(cpos+cneg)) . A resulting forest plot is shown in Figure 2 (left plot). The parameter estimates before back-transformation are $\hat{\mu }=-0.71$ and $\hat{\tau }=0.56$ . Using the integral back-transformation, the estimated mean effect size and its CI are 0.57 [0.40, 0.81], while the results obtained applying $g^{-1}(=\exp)$ are 0.49 [0.34, 0.70].

In principle, the treatment comparison can also be defined inversely. The right panel of Figure 2 shows the forest plot for the same data set using inversely defined risk ratios RR=(cpos/(cpos+cneg))/(tpos/(tpos+tneg)) . The raw estimates and CI bounds as well as the absolute transformation difference increase largely because of the asymmetrical nature of the transformation or equivalently the skewness of the log-normal distribution. The results using the integral back-transformation are 2.39 [1.68, 3.40], while those using the inverse back-transformation are 2.04 [1.44, 2.91].

Importantly, this numerical example clearly demonstrates that the integral back-transformation is not invariant to a reversal of the comparison direction, such that exchanging treatment and control does not generally lead to reciprocal effect estimates. In the forest plot, this is reflected by the fact that the red diamond is the only element that is not mirrored at the vertical line at one. To further illustrate the relevance of this issue, the following section presents an additional example in which extreme heterogeneity amplifies this problem.

### 4.2 Detailed case study on *log* transformations for Kearon et al.

We re-analyzed an additional meta-analysis containing 2×2 contingency tables by Kearon et al.^16^ on the precision of venous ultrasound for the diagnosis of deep venous thrombosis. The results indicate an extreme level of heterogeneity. As a result, the transformation bias is larger, and additionally, inconsistent CI results are obtained. A detailed investigation of the sources of heterogeneity and a medical justification for conducting a meta-analysis on these heterogeneous data is not within the scope of this re-analysis.

We denote the deep venous thrombosis cases and non-cases based on the contrast venography classification as: true positives (“tp”), false positives (“fp”), true negatives (“tn”), and false negatives (“fn”). In our case study, a *log* transformation is applied to the diagnostic ORs, which compare the odds of a correct classification for positive cases with those for negative cases. The OR is defined as OR=(tp⋅fn)/(fp⋅tn) . There are two studies with, in relation, extremely large ORs (Schindler et al. with OR = 30.39 and Mattos et al. with OR = 4.71). This leads to a large and imprecise estimate of $\hat{\tau }=1.76$ . As discussed in Section 3, this large and imprecisely estimated value has a drastic impact on the integral back-transformation.

A corresponding forest plot can be found in Figure 3 (left plot). The results of the two back-transformations differ strongly: 0.11 [0.05, 0.23] for the standard inverse back-transformation and 0.53 [0.25, 1.08] for the integral one. Since the CI contains “1” only in the integral transformation case, this would even lead to a different test decision when interpreting the CIs as a test for the mean OR for venography classification accuracy for cases versus non-cases (the inverse back-transformation and CI for the median confirm the effect, the integral back-transformation and CI for the mean do not).

When using the inverse definition of the OR ( OR=(fp⋅tn)/(tp⋅fn) ), the difference between the estimates of the two back-transformation methods becomes even larger. The results are visualized in the right plot of Figure 3 (8.89 [4.32, 18.31] for the inverse back-transformation and 41.51 [20.15, 85.53] for the integral back-transformation). In this case, both CIs confirm the effect. For the $g^{-1}$ transformation, this is consistent with the result based on the inverse OR definition (left plot). However, for the integral transformation, which corresponds to the correct test for the back-transformed mean, the problem described in Section 3 arises. Due to the one-sided bias and the skewness of the back-transformed distribution, the test, based on the back-transformed CI, differs when using the inverse OR.

Even though there is often a prescriptive direction used in the definition of the odds or risk ratio (i.e., intervention vs. comparator as recommended in Chapter 6.4 of the Cochrane Handbook^17^), this inconsistency is alarming. This is severe especially in test decisions but also for regular point estimates. As noted at the end of Section 3, we therefore recommend performing all tests on the transformed scale or use the standard back-transformation and then conduct a test for the back-transformed median (and interpret it as such). Thus, hypothesis tests for the back-transformed mean should not be conducted, and CIs should only be reported when they are explicitly required.

### 4.3 Overview of case studies for different types of transformations

An overview of the results when applying the two *log* transformations from the previous sections and the other transformations for several additional case studies can be found in Table 2. The corresponding effect measures and transformations are indicated. In addition, estimates of $\hat{\mu }$ and $\hat{\tau }$ are reported. The corresponding forest plots can be found in Section B of the Supplementary Material.

**Table 2:** Re-analyzed data sets with different effect measures and transformations with results separated for the standard inverse and the integral back-transformation Table 2 Long description.

					Inverse back-transf.		Integral back.-transf.	
Study	Measure	Transf.	$\hat{\mu }$	$\hat{\tau }$	Point est.	CI	Point est.	CI
Colditz et al.^15^	Risk ratios	log	− 0.71	0.56	0.49	[0.34, 0.70]	0.57	[0.40, 0.81]
	Inv. risk ratios	log	0.71	0.56	2.04	[1.44, 2.91]	2.39	[1.68, 3.40]
Kearon et al.^16^	Odds ratios	log	− 2.18	1.76	0.11	[0.05, 0.23]	0.53	[0.25, 1.08]
	Inv. odds ratio	log	2.18	1.76	8.89	[4.32, 18.31]	41.51	[20.15, 85.53]
de Bruin et al.^18^	Proportions	logit	− 0.11	0.63	0.47	[0.38, 0.57]	0.47	[0.39, 0.56]
		probit	− 0.07	0.39	0.47	[0.38, 0.57]	0.47	[0.39, 0.56]
		arcsine	0.76	0.15	0.47	[0.38, 0.57]	0.48	[0.39, 0.56]
Pritz et al.^19^	Proportions	logit	1.14	0.60	0.76	[0.66, 0.83]	0.74	[0.65, 0.82]
		probit	0.74	0.39	0.77	[0.68, 0.85]	0.76	[0.66, 0.83]
		arcsine	1.12	0.21	0.81	[0.70, 0.90]	0.78	[0.69, 0.86]
Credé et al.^21^	Correlations	*r*-to-*z*	0.37	0.24	0.36	[0.28, 0.43]	0.34	[0.26, 0.41]
Demir et al.^22^	α coefficients	AHW	0.35	0.01	0.73	[0.68, 0.77]	0.71	[0.66, 0.76]
		ABT	1.33	0.48	0.74	[0.69, 0.78]	0.71	[0.65, 0.75]

Proportions are analyzed in two studies (de Bruin et al.^18^ and Pritz et al.^19^). The first study summarizes results from control groups in trials investigating antiviral therapies. The proportion of patients with an undetectable viral load in standard care groups is used for this meta-analysis. The second study includes research on the effectiveness of hyperdynamic therapy for treating cerebral vasospasm. The data set, originally compiled by Pritz et al.,^19^ is available through Zhou et al.^20^ The proportions analyzed refer to the share of patients who showed improvement following the therapy. We applied the *logit*, *probit*, and *arcsine* transformations to both data sets. A larger transformation effect was observed for Pritz et al.^19^ due to the different size of $\hat{\mu }$ .

An example of the application of *Fisher’s r-to-z* transformation on correlation coefficients is provided using the data set from Credé et al.^21^ This analysis explores the relationship between class attendance and grade point average among college students. Furthermore, the *AHW* and *ABT* transformations are applied to the data set from Demir et al.,^22^ which investigates the reliability of the Mother-to-Infant Bonding Scale. Reliability is assessed using the reported coefficients of Cronbach’s α . The results indicate that the transformation bias was more pronounced in the case of the *ABT* transformation.

## 5 Summary and discussion

Meta-analyses commonly involve transformations of effect sizes to approximate a normally distributed sampling distribution. These transformations are typically followed by back-transformations to return the results to the original scale for easier interpretation. However, the standard approach, applying the inverse of the transformation function, yields the median of the distribution of effects on the back-transformed scale rather than its mean.

The first key message of this article is therefore conceptual. Interpreting the result of a standard inverse back-transformation as a mean effect size is incorrect. While the use of the inverse transformation is not inappropriate, the resulting estimate must be explicitly interpreted and reported as a median effect size, representing the typical value of the distribution of true effects.

The second key message concerns estimation of the mean on the original scale. We presented an integral back-transformation aiming for this mean. We generalized this approach to arbitrary effect size transformations and proposed a modification that is also valid for transformations with a bounded range. While this approach is methodologically correct in targeting the mean, it entails several issues and must be applied with caution. These issues are sensitivity to the estimation of $\tau^{2}$ and the occurrence of inverse results for inversely defined effect sizes. This lack of invariance is present when the transformation is not point-symmetric around an effect-size-specific neutral value.

The discrepancy between standard inverse and integral back-transformations depends on both the shape of the transformation function and the degree of between-study heterogeneity, as quantified by $\tau^{2}$ . This relationship is illustrated in this article across several effect size measures and transformations. In particular, transformation difference can be substantial for transformation functions with unbounded domains, such as the *log* transformation applied to ratio measures.

The integral back-transformation not only enables estimation of the mean effect size on the original scale, but also provides a formally valid basis for conducting hypothesis tests targeting this mean. Such tests can be defined by applying the integral back-transformation directly to the CI bounds on the transformed scale. However, we generally recommend performing statistical tests on the transformed scale, where the normality assumption for the distribution of true effects is originally specified. Equivalent test results are obtained from CIs for the back-transformed median, which can be constructed using the standard inverse transformation applied to the confidence limits on the transformed scale.

We emphasize that, even if the mean effect size on the original scale is the primary target, directly averaging the untransformed study-specific estimates is generally neither equivalent nor advisable. The rationale for working on a transformed scale is to better satisfy model assumptions and thereby ensure statistically sound estimation and inference. In particular, transformations are chosen such that both the distribution of true effects and the corresponding sampling distributions are closer to normality, while also stabilizing the variance across studies. The integral back-transformation should therefore be understood as a way to recover the mean effect on the original scale after fitting the model under these more appropriate conditions.

For transformations where the range of g is not equal to R ( V⊊R , e.g., for the *arcsine* transformation), the integral transformation cannot be applied directly since the probability distribution of the fitted normal distribution extends V . If this issue is ignored, formally, a truncated normal distribution is back transformed. We describe this truncation-based approach and introduce a corresponding integral back-transformation. The truncation approach generally leads to biased results. A possible alternative, that is potentially less biased, is clipping, where the irregular probability mass is assigned as point mass to the respective boundary of V . However, it must be noted that this also implies a different formal distributional assumption.

In addition, it should be noted that models assuming other distributions for the true effects^23^^,^ ^24^ are equally affected by the issues discussed in the present article. In this case, the normal density in Equation (2.3) needs to be replaced with whatever distribution is assumed for the true effects. Moreover, models using “exact likelihoods” (e.g., when modeling the counts of 2×2 tables via binomial and Poisson distributions) also typically assume a (normal) distribution of true effects on a transformed scale.^25^^,^ ^26^ Therefore, to obtain the mean of the untransformed distribution, the integral back-transformation should also be used.

This article describes the issue in the context of frequentist statistics. A similar principle naturally applies in a Bayesian setting.^27^ When the mean of the posterior samples is computed, it can be back-transformed using either back-transformation. Applying the inverse transformation yields the median. Using the integral transformation in combination with a variance estimate results in the mean. This is identical to directly back-transforming the posterior samples and subsequently computing the median or mean on the original scale.

## 6 Practical guidance

Importantly, the choice between the two back-transformations should be guided by the analyst’s underlying goal. When the objective of a meta-analysis is primarily descriptive, and interest focuses on a central or typical effect, the median effect size obtained via the standard inverse back-transformation is often preferable. This approach is comparatively robust in the presence of strong skewness on the original scale and substantial between-study heterogeneity. Moreover, it is less sensitive to imprecision in the estimation of the between-study variance parameter $\tau^{2}$ as the corresponding back-transformation function is independent of it.

In contrast, the integral back-transformation is particularly important when the target estimand is explicitly defined as a mean effect size. This includes reporting strategies that require mean effects by definition, comparisons with primary or secondary studies reporting means on the original scale, and applications in which the meta-analytic result is used as input for further linear calculations. Examples for this are additive projections, such as determining expected event counts based on pooled proportions or extrapolating average treatment costs. Similarly, secondary statistical analyses and decision-theoretic frameworks that rely on optimality properties defined in terms of expectations necessitate the use of a mean-based back-transformation. Thus, from a practical perspective, the estimand should be chosen deliberately and reported transparently. In cases of doubt, it is safer to use the standard inverse transformation and to correctly report the resulting estimate as a median.

## Supporting information

10.1017/rsm.2026.10096.sm001 Igelmann et al. supplementary material Igelmann et al. supplementary material

## Data Availability

All materials, including the Supplementary Material document, the R scripts for reproducing the analyses and examples, as well as all referenced figures, are openly available via the repository at https://doi.org/10.17877/TUDODATA-2026-7UFBJF. The datasets used for the analysis are accessible through the R package metadat.^13^

## Supplementary material

The supplementary material for this article can be found at https://doi.org/10.1017/rsm.2026.10096.

## Long descriptions

### Table 1 Long description

The table contains 6 columns and 7 rows of data.

Column headers:

1. Transformation.

2. g ( x ).

3. g inverse ( y ).

4. D yields V.

5. psi sub g in m e t a f o r.

6. Applications.

Data rows:

- log: g ( x ) is log ( x ); g inverse ( y ) is exp ( x ); D yields V is ( 0, infinity ) yields R; m e t a f o r function is transf.exp.int; Applications are Ratios (odds, risk, ...).

- logit: g ( x ) is log of the fraction x over 1 minus x; g inverse ( y ) is 1 over 1 plus e to the power of minus x; D yields V is ( 0, 1 ) yields R; m e t a f o r function is transf.ilogit.int; Applications are Proportions.

- probit: g ( x ) is Phi super minus 1 ( x ); g inverse ( y ) is Phi ( x ); D yields V is ( 0, 1 ) yields R; m e t a f o r function is transf.iprobit.int; Applications are probabilities.

- arcsine: g ( x ) is arcsin ( square root of x ); g inverse ( y ) is sin squared ( x ); D yields V is [ 0, 1 ] yields [ 0, pi / 2 ]; m e t a f o r function is transf.iarcsin.int.

- Fisher's r-to-z: g ( x ) is artanh ( x ); g inverse ( y ) is tanh ( x ); D yields V is ( minus 1, 1 ) yields R; m e t a f o r function is transf.ztor.int; Applications are Correlations.

- A H W: g ( x ) is 1 minus ( 1 minus x ) to the 1 / 3 power; g inverse ( y ) is 1 minus ( 1 minus x ) cubed; D yields V is [ 0, 1 ] yields [ 0, 1 ]; m e t a f o r function is transf.iahw.int; Applications are Alpha coefficients.

- A B T: g ( x ) is minus log ( 1 minus x ); g inverse ( y ) is 1 minus exp ( minus x ); D yields V is [ 0, 1 ) yields [ 0, infinity ); m e t a f o r function is transf.iabt.int.

Note: Phi is the cumulative distribution function of the standard normal distribution.

Navigate back to Table 1.

### Figure 1 Long description

The image consists of two side-by-side line graphs. A shared legend on the right indicates four line styles corresponding to tau hat values: dotted for 0.05, dash-dot for 0.1, dashed for 0.25, and solid for 0.5.

Left Panel: Log transformation.

* The primary x-axis at the bottom is the log-O R / R R scale: mu hat, ranging from -2 to 2.

* A secondary x-axis at the top shows the O R / R R scale: exp(mu hat) with values 0.14, 0.37, 1, 2.72, and 7.39.

* The y-axis is labeled transformation difference, ranging from 0.0 to 1.5.

* All four curves start near zero on the left. As mu hat increases, the curves rise exponentially. The solid line (tau hat 0.5) shows the steepest increase, reaching above 1.5, while the dotted line (tau hat 0.05) remains nearly flat near zero.

Right Panel: r-to-z transformation.

* The primary x-axis at the bottom is the Fisher-z scale: mu hat, ranging from -2 to 2.

* A secondary x-axis at the top shows the r scale: tanh(mu hat) with values -0.96, -0.76, 0, 0.76, and 0.96.

* The y-axis is labeled transformation difference, ranging from -0.05 to 0.05.

* The curves exhibit an S-shaped or sinusoidal pattern centered at (0,0). For negative mu hat values, the curves peak positively, and for positive mu hat values, they dip negatively. The solid line (tau hat 0.5) has the highest amplitude, peaking near 0.08 and dipping to -0.08, while the dotted line (tau hat 0.05) shows minimal deviation from the zero line.

Navigate back to Figure 1.

### Figure 2 Long description

A multi-panel figure containing two forest plots side-by-side, both titled Colditz et al. 1994. Each plot has columns for Author s and Year, a graphical representation of Risk Ratio R R with 95 percent Confidence Intervals C I on a log scale, and a numerical column for R R [95 percent C I].

Left Panel: Vaccinated over Control.

* The list of 13 studies includes Aronson 1948, Ferguson and Simes 1949, Rosenthal et al 1960, Hart and Sutherland 1977, Frimodt-Moller et al 1973, Stein and Aronson 1953, Vandiviere et al 1973, T P T Madras 1980, Coetzee and Berjak 1968, Rosenthal et al 1961, Comstock et al 1974, Comstock and Webster 1969, and Comstock et al 1976.

* Most studies show an R R less than 1, indicating a protective effect, with point estimates ranging from 0.20 to 1.56.

* At the bottom, two summary diamonds are shown. The g super minus 1 back-transformation diamond is black with a value of 0.49 [0.34, 0.70]. The integral back-transformation diamond is red with a value of 0.57 [0.40, 0.81].

Right Panel: Control over Vaccinated.

* This panel presents the reciprocal values of the left panel for the same 13 studies.

* Most studies show an R R greater than 1, with point estimates ranging from 0.64 to 5.06.

* The summary diamonds at the bottom show the g super minus 1 back-transformation at 2.04 [1.44, 2.91] in black, and the integral back-transformation at 2.39 [1.68, 3.40] in red.

The x-axis for both plots is labeled Risk Ratio log scale with tick marks at 0.05, 0.25, 1, and 4 for the left, and 0.25, 1, 2.5, and 10 for the right. A vertical dashed line indicates the null effect at R R equals 1.

Navigate back to Figure 2.

### Figure 3 Long description

A two-panel forest plot. Each panel contains a list of 28 studies by Author and Year, a graphical representation of Odds Ratios with 95 percent C I, and a numerical column for Odds Ratio values.

Left Panel: Positive over negative patients.

- The x-axis is a log scale for Odds Ratio with markers at 0.01, 0.15, 1, and 5. A vertical dashed line is at 1.

- Individual studies range from Elias et al. 1987 at the top to Atri et al. 1996 at the bottom. Most studies show Odds Ratios below 1, with horizontal error bars and square point estimates. Some error bars have arrows indicating they extend beyond the visible scale.

- At the bottom, two summary diamonds are shown: g super minus 1 back-transformation is a black diamond at 0.11 [0.05, 0.23]; integral back-transformation is a red diamond at 0.53 [0.25, 1.08].

Right Panel: Negative over positive patients.

- The x-axis is a log scale for Odds Ratio with markers at 0.1, 1, 20, and 90. A vertical dashed line is at 1.

- The studies are identical to the left panel but show inverted results. Most studies show Odds Ratios above 1.

- At the bottom, the summary diamonds are: g super minus 1 back-transformation is a black diamond at 8.89 [4.32, 18.31]; integral back-transformation is a red diamond at 41.51 [20.15, 85.53].

Navigate back to Figure 3.

### Table 2 Long description

The table consists of 9 columns. The primary headers are Study, Measure, Transf., mu hat, tau hat, Inverse back-transf. (sub-divided into Point est. and C I), and Integral back-transf. (sub-divided into Point est. and C I).

* Colditz et al. (Risk ratios, log): mu hat -0.71, tau hat 0.56. Inverse: 0.49 [0.34, 0.70]. Integral: 0.57 [0.40, 0.81].

* Colditz et al. (Inv. risk ratios, log): mu hat 0.71, tau hat 0.56. Inverse: 2.04 [1.44, 2.91]. Integral: 2.39 [1.68, 3.40].

* Kearon et al. (Odds ratios, log): mu hat -2.18, tau hat 1.76. Inverse: 0.11 [0.05, 0.23]. Integral: 0.53 [0.25, 1.08].

* Kearon et al. (Inv. odds ratio, log): mu hat 2.18, tau hat 1.76. Inverse: 8.89 [4.32, 18.31]. Integral: 41.51 [20.15, 85.53].

* de Bruin et al. (Proportions):

- logit: mu hat -0.11, tau hat 0.63. Inverse: 0.47 [0.38, 0.57]. Integral: 0.47 [0.39, 0.56].

- probit: mu hat -0.07, tau hat 0.39. Inverse: 0.47 [0.38, 0.57]. Integral: 0.47 [0.39, 0.56].

- arcsine: mu hat 0.76, tau hat 0.15. Inverse: 0.47 [0.38, 0.57]. Integral: 0.48 [0.39, 0.56].

* Pritz et al. (Proportions):

- logit: mu hat 1.14, tau hat 0.60. Inverse: 0.76 [0.66, 0.83]. Integral: 0.74 [0.65, 0.82].

- probit: mu hat 0.74, tau hat 0.39. Inverse: 0.77 [0.68, 0.85]. Integral: 0.76 [0.66, 0.83].

- arcsine: mu hat 1.12, tau hat 0.21. Inverse: 0.81 [0.70, 0.90]. Integral: 0.78 [0.69, 0.86].

* Crede et al. (Correlations, r-to-z): mu hat 0.37, tau hat 0.24. Inverse: 0.36 [0.28, 0.43]. Integral: 0.34 [0.26, 0.41].

* Demir et al. (alpha coefficients):

- A H W: mu hat 0.35, tau hat 0.01. Inverse: 0.73 [0.68, 0.77]. Integral: 0.71 [0.66, 0.76].

- A B T: mu hat 1.33, tau hat 0.48. Inverse: 0.74 [0.69, 0.78]. Integral: 0.71 [0.65, 0.75].

Navigate back to Table 2.
