# Supplementary material for: Back-transformations in random-effects meta-analysis—Impact and interpretation
Source: Res Synth Methods. 2026 May 15;17(6):1261–74. doi: 10.1017/rsm.2026.10096 (PMC13732378; doi:10.1017/rsm.2026.10096)
Supplement: Supplementary file 1 [file S1759287926100969sup001.pdf]

# Supplement to: Back-Transformations in Random-Effects Meta-Analysis - Impact and Interpretation

Jan-Bernd Igelmann[1], Markus Pauly[1,2] Wolfgang Viechtbauer[3]

This document supplements the main paper "Back-Transformations in Random-Effects Meta-Analysis - Impact and Interpretation". Section A is an addition to Section 3 of the main paper. The transformation difference is visualized for all further transformations (*logit*, *probit*, *arcsine*, *AHW*, and *ABT*). Section B expands on Section 4.3 of the main paper, presenting all additional forest plots of the application examples discussed in that section.

## Contents

**A Visualized Transformation Difference** **2**
A.1 Transformation Difference: logit, probit, and arcsine . . . . . 2
A.2 Transformation Difference: AHW und ABT . . . . . 4
**B Forest Plots for Applications Examples** **5**
B.1 de Bruin et al. . . . . . 5
B.2 Pritz et al. . . . . . 8
B.3 Credé et al. . . . . . 11
B.4 Demir et al. . . . . . 12

[1]Department of Statistics, TU Dortmund University
[2]Research Center Trustworthy Data Science and Security, UA Ruhr, Germany
[3]Department of Psychiatry and Neuropsychology, Maastricht University, The Netherlands

## A. Visualized Transformation Difference

### *A.1. Transformation Difference: logit, probit, and arcsine*

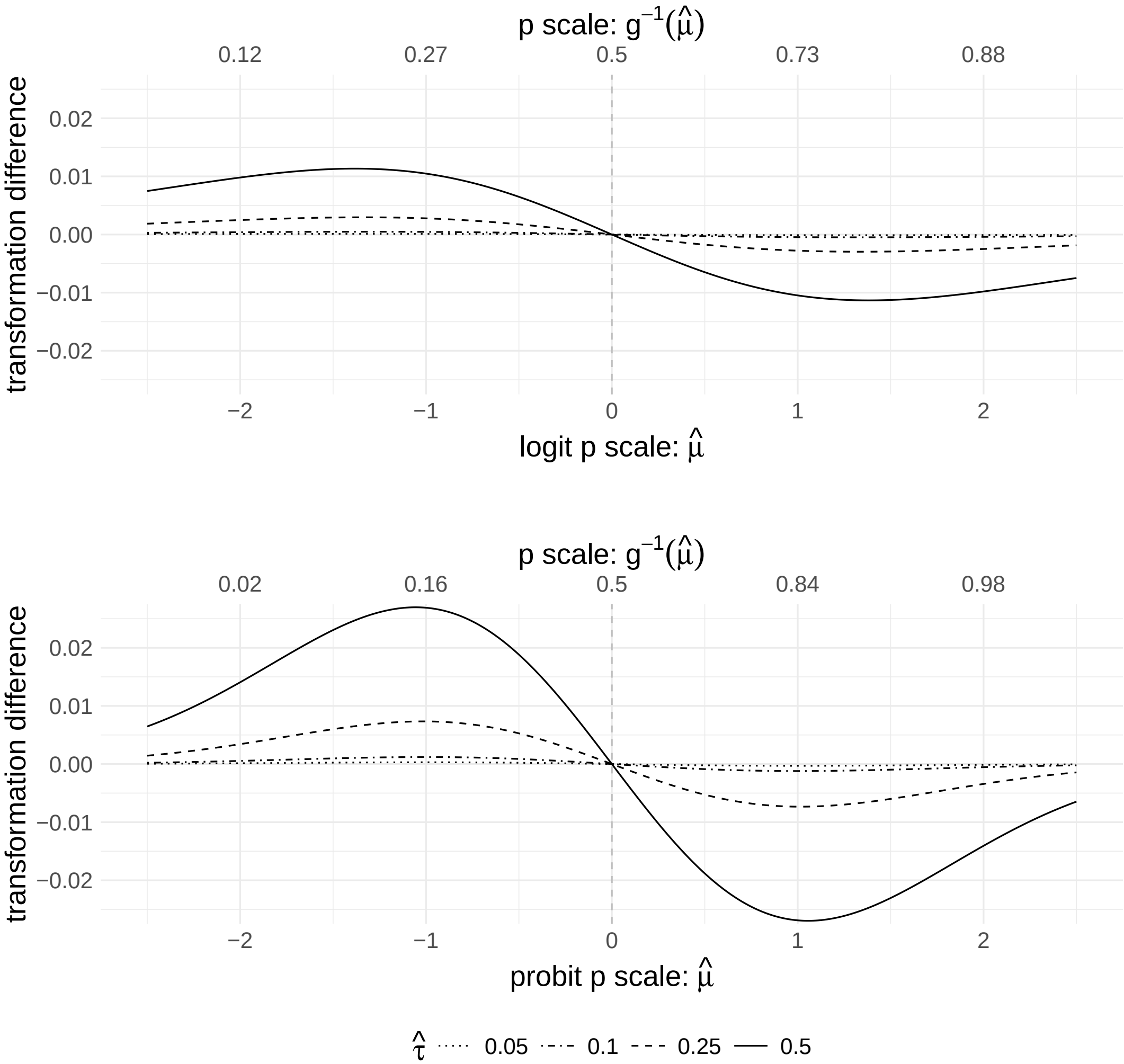


***Figure 1.*** *Visualization of the transformation difference $\psi_g(\hat{\mu}, \hat{\tau}^2) - g^{-1}(\hat{\mu})$ for the logit transformation (top) and probit transformation (bottom).*

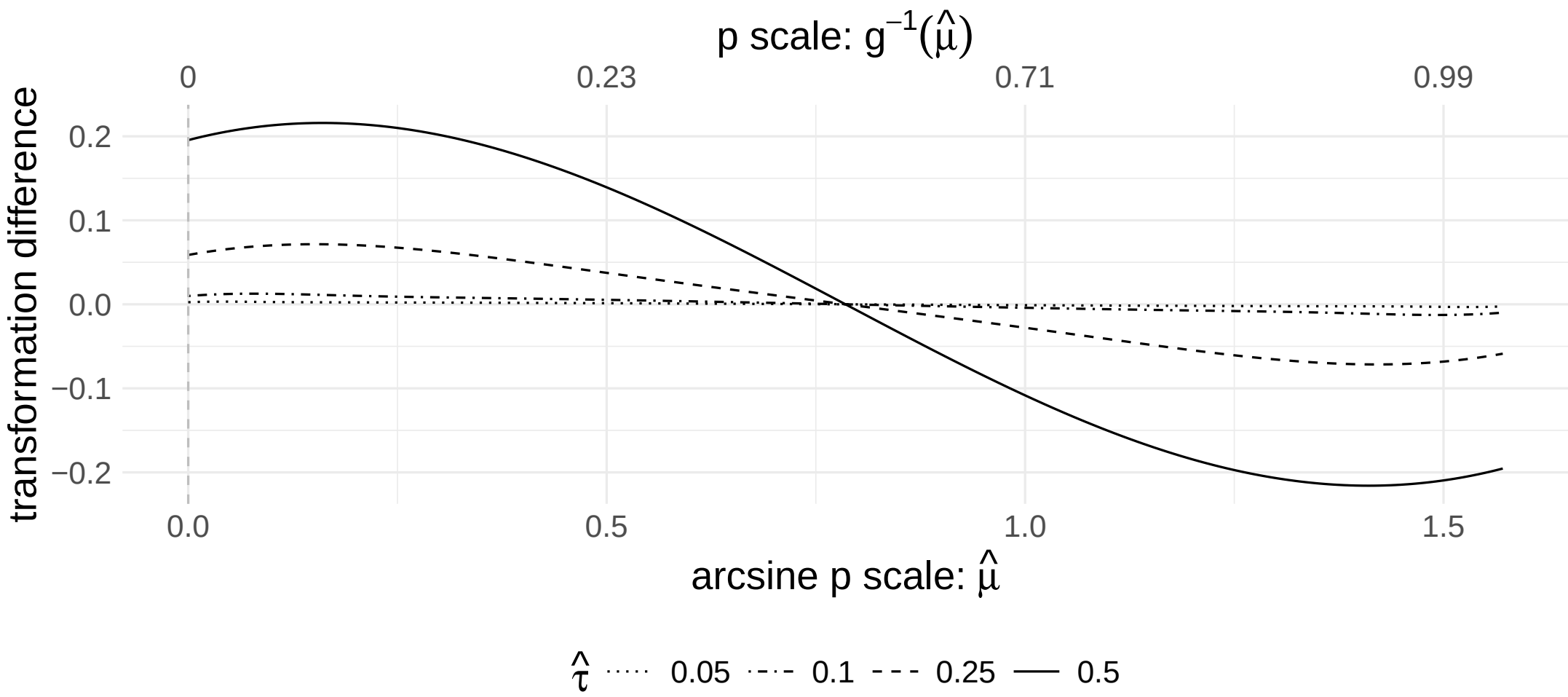


***Figure 2.*** *Visualization of the transformation difference* $\psi_{arcsine}(\hat{\mu}, \hat{\tau}^2) - \sin^2(\hat{\mu})$ *for the arcsine transformation.*

### *A.2. Transformation Difference: AHW und ABT*

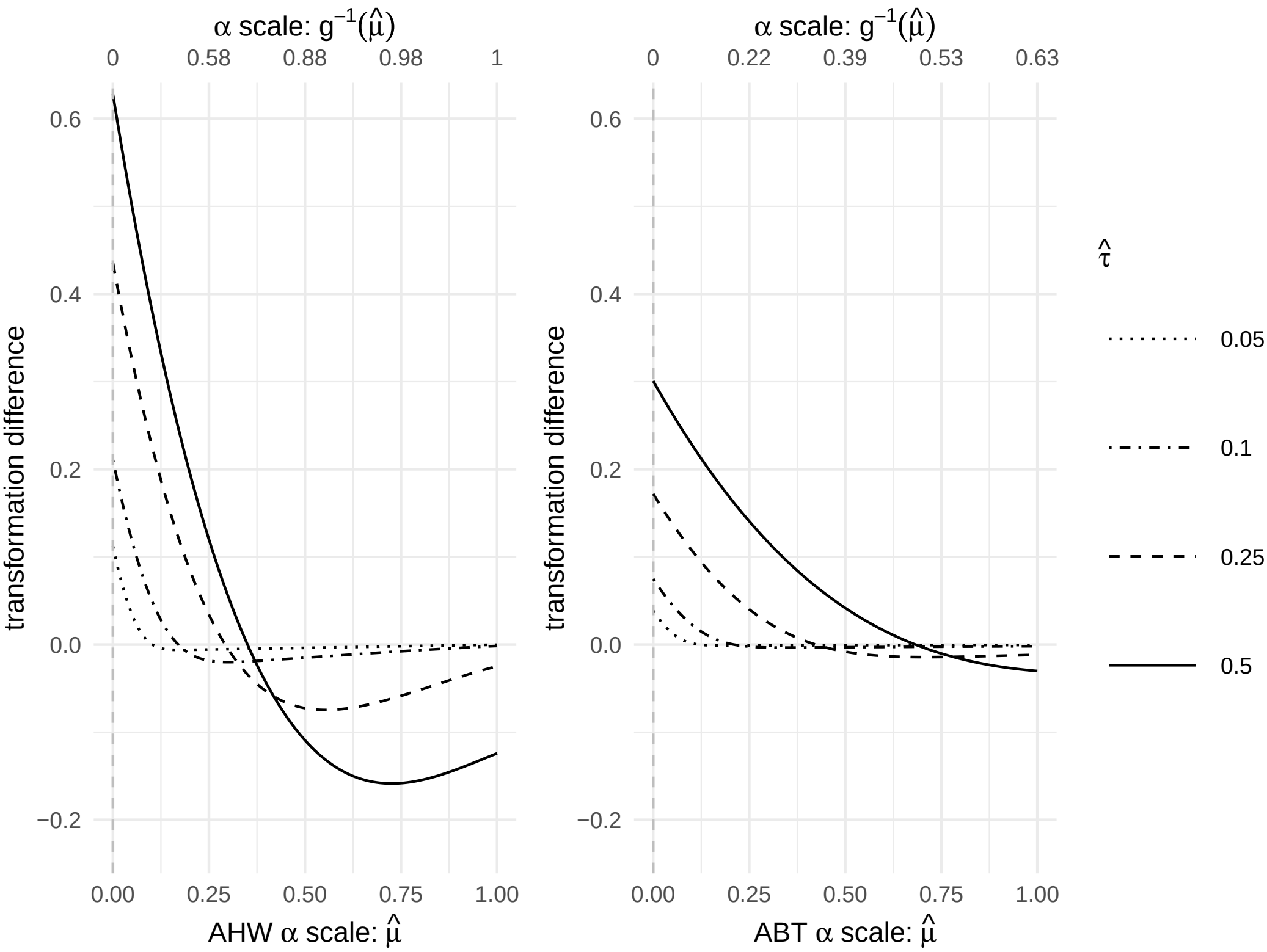


***Figure 3.*** *Visualization of the transformation difference $\psi_g(\hat{\mu}, \hat{\tau}^2) - g^{-1}(\hat{\mu})$ for the AHW transformation (left) and ABT transformation (right).*

## B. Forest Plots for Applications Examples

### *B.1. de Bruin et al.*

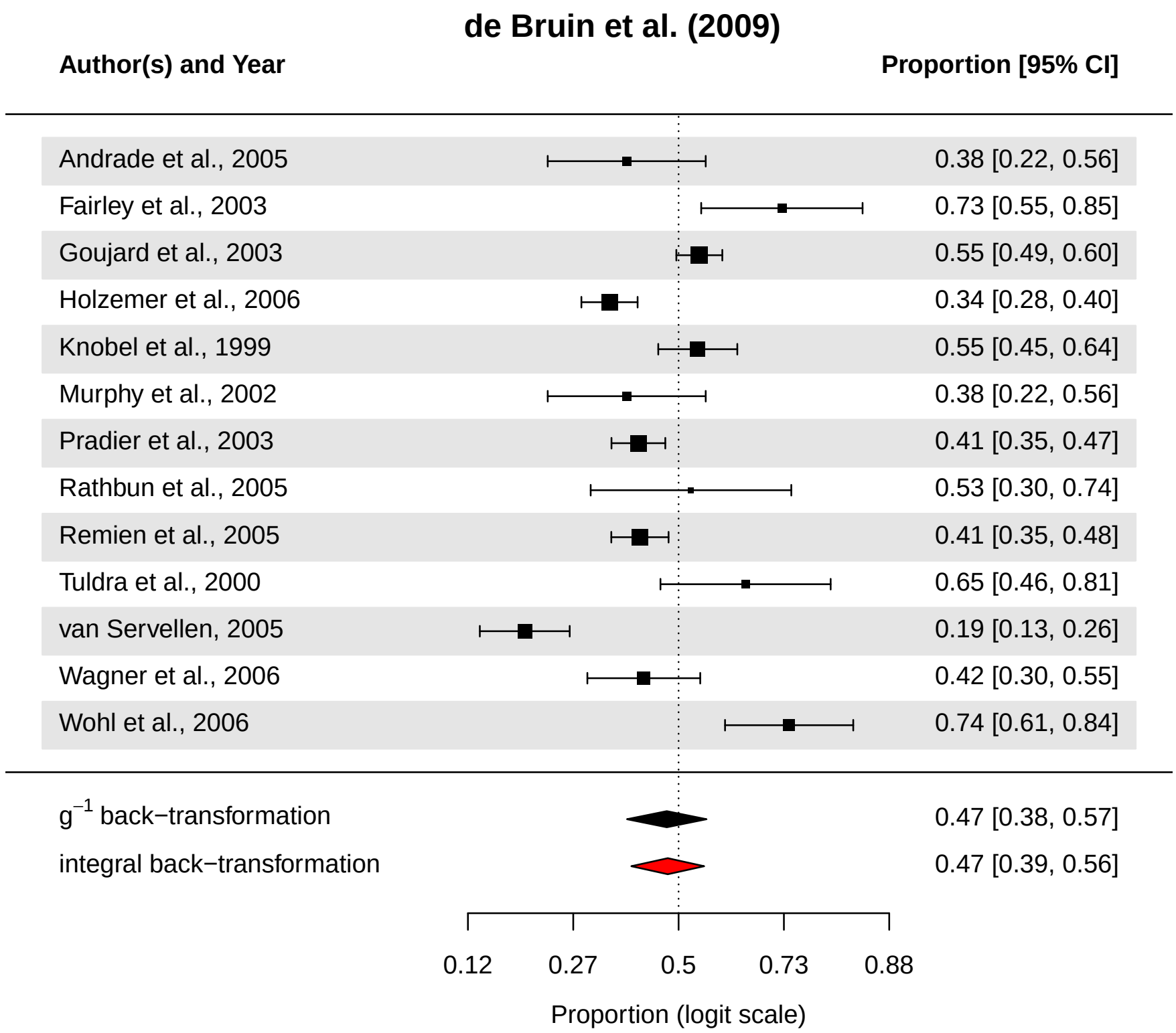


***Figure 4.*** *Forest plot of the de Bruin et al. meta-analysis*[1] *with* $g^{-1}$ *and integral back-transformation for the logit transformation applied on proportions;* $\hat{\tau} = 0.63$.

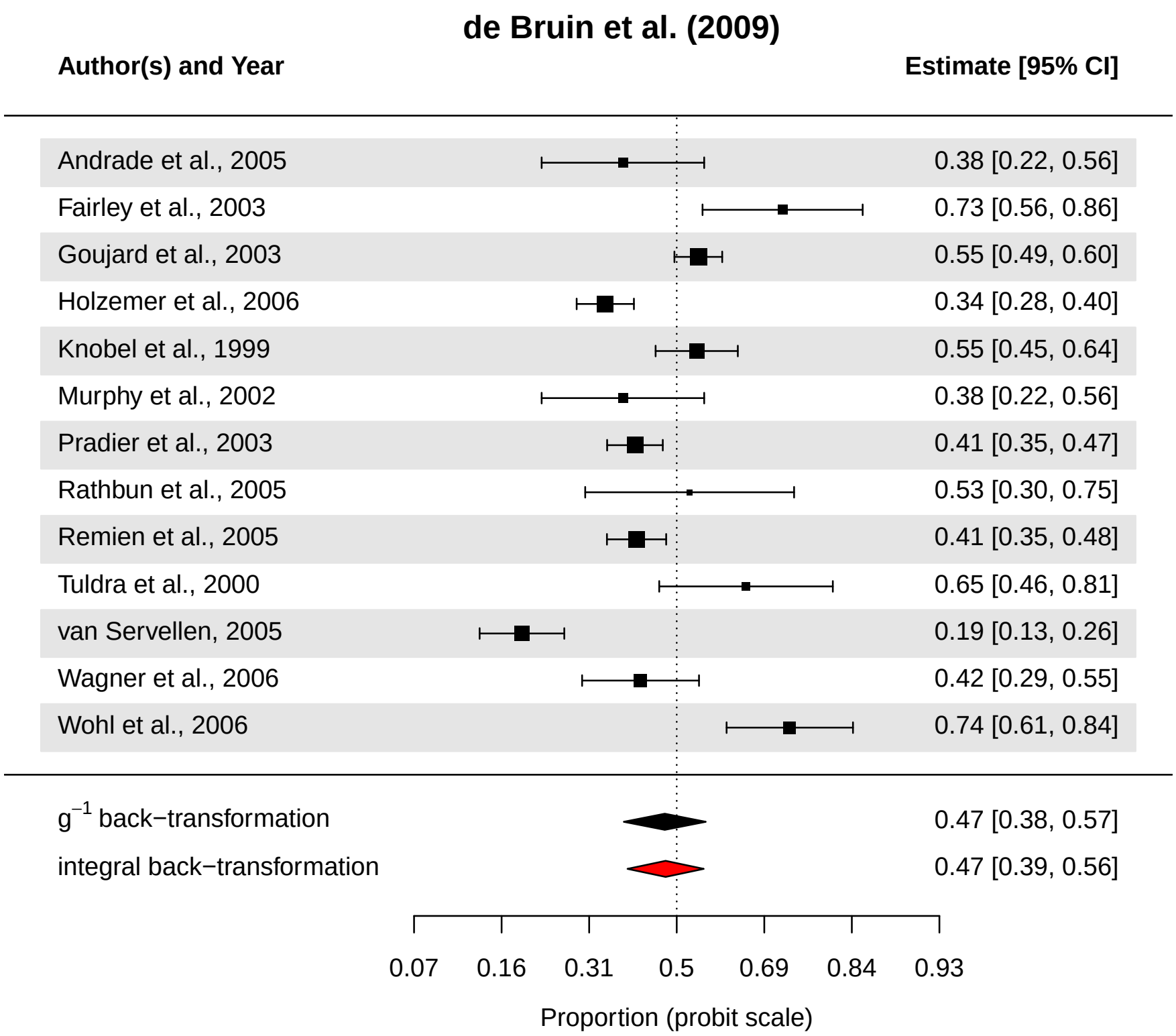


***Figure 5.*** *Forest plot of the de Bruin et al. meta-analysis*[1] *with* $g^{-1}$ *and integral back-transformation for the probit transformation applied on proportions;* $\hat{\tau} = 0.39$.

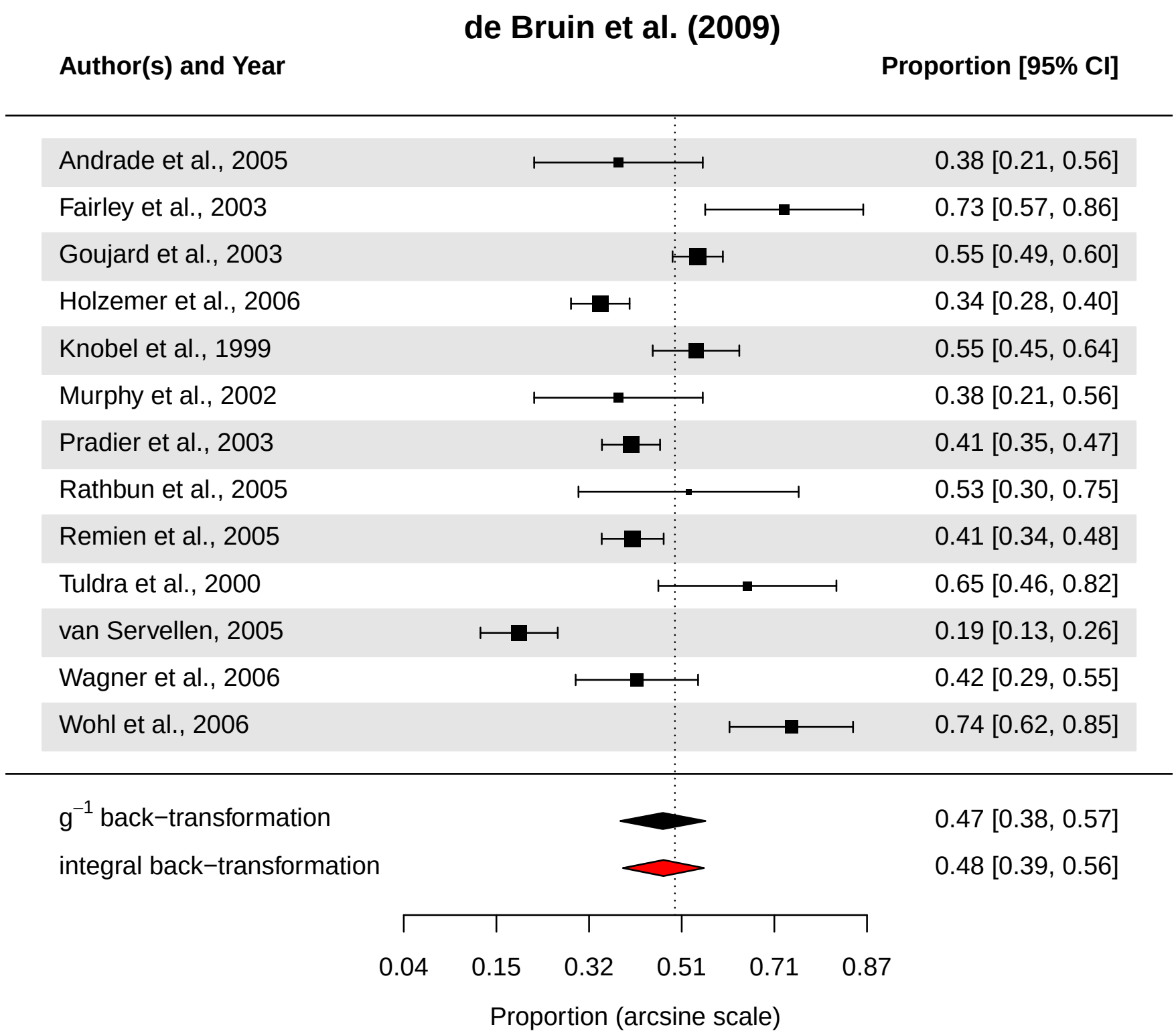


***Figure 6.*** *Forest plot of the de Bruin et al. meta-analysis*[1] *with* $g^{-1}$ *and integral back-transformation for the arcsine transformation applied on proportions;* $\hat{\tau} = 0.15$.

### *B.2. Pritz et al.*

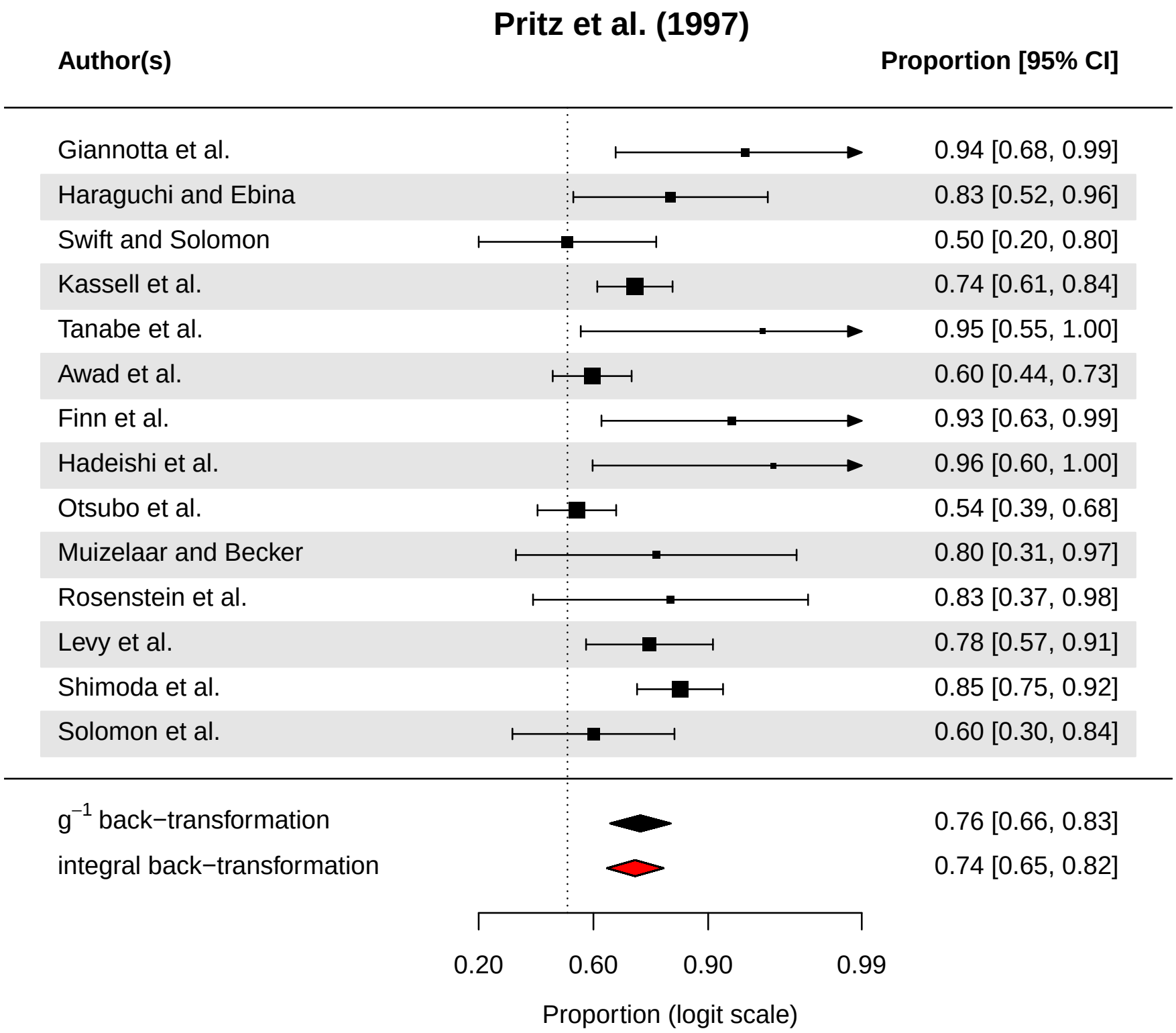


***Figure 7.*** *Forest plot of the Pritz et al. meta-analysis*[2] *with* $g^{-1}$ *and integral back-transformation for the logit transformation applied on proportions;* $\hat{\tau} = 0.60$.

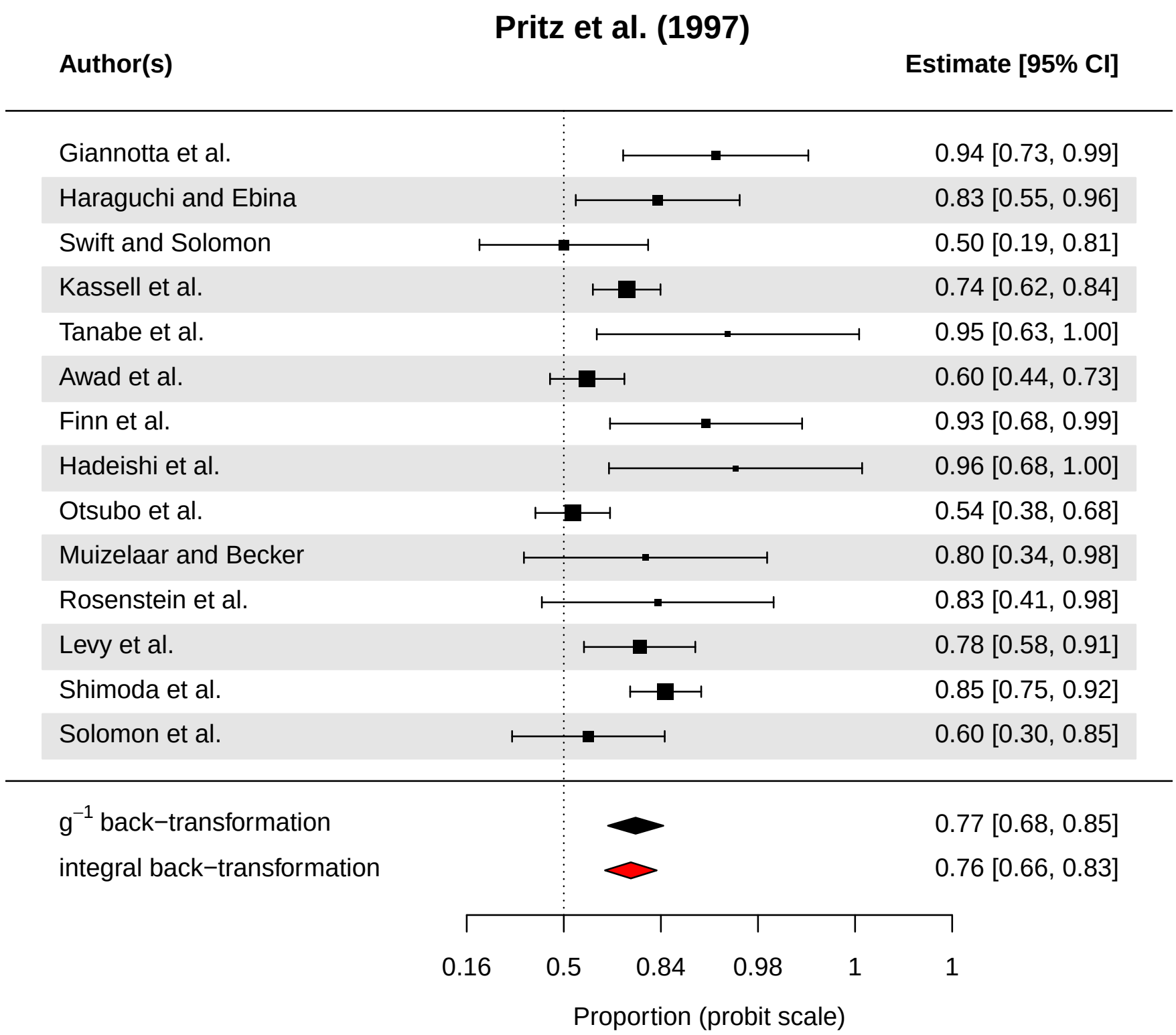


***Figure 8.*** *Forest plot of the Pritz et al. meta-analysis*[2] *with* $g^{-1}$ *and integral back-transformation for the probit transformation applied on proportions;* $\hat{\tau} = 0.39$.

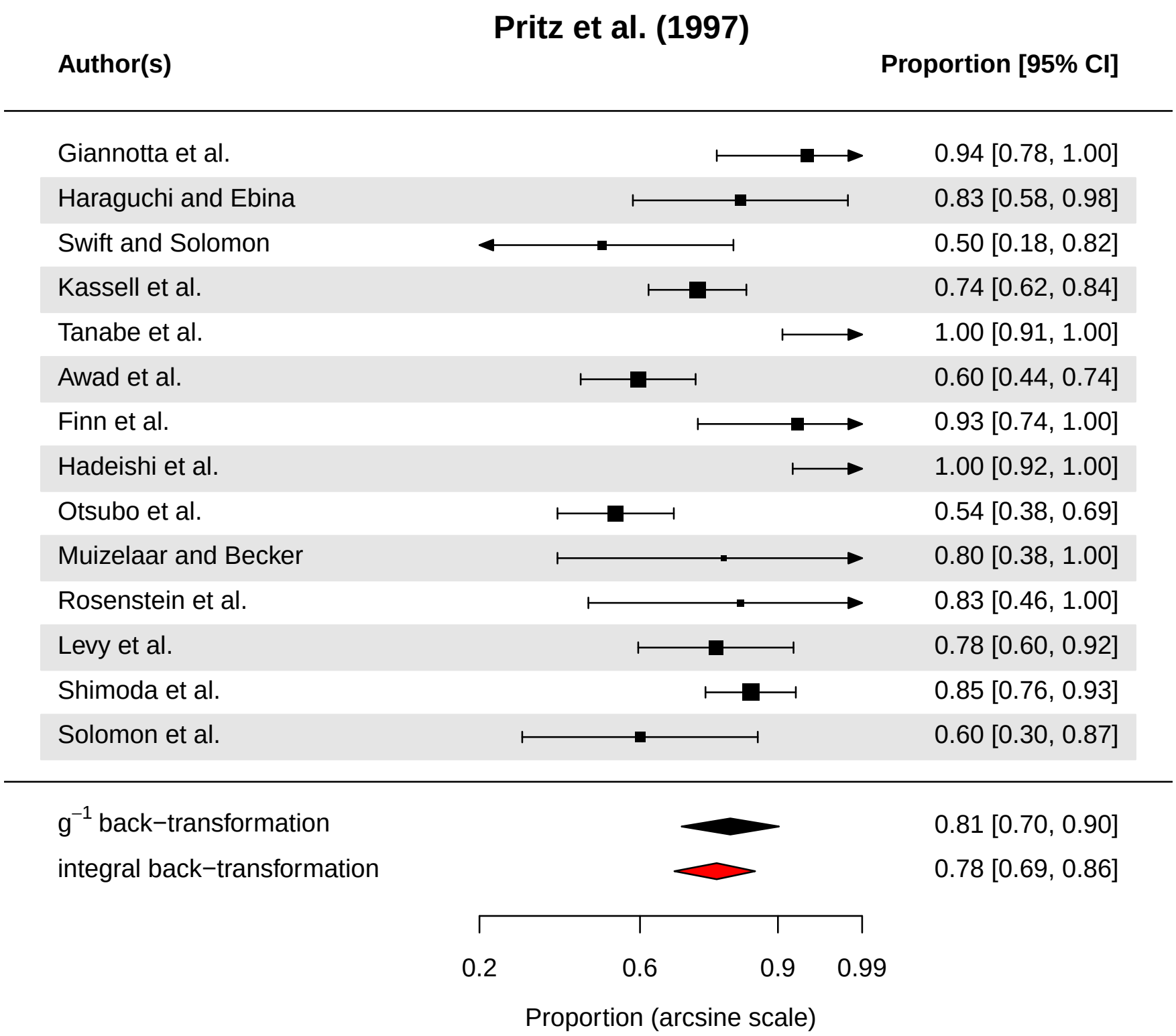


***Figure 9.*** *Forest plot of the Pritz et al. meta-analysis*[2] *with* $g^{-1}$ *and integral back-transformation for the arcsine transformation applied on proportions;* $\hat{\tau} = 0.21$.

### *B.3. Credé et al.*

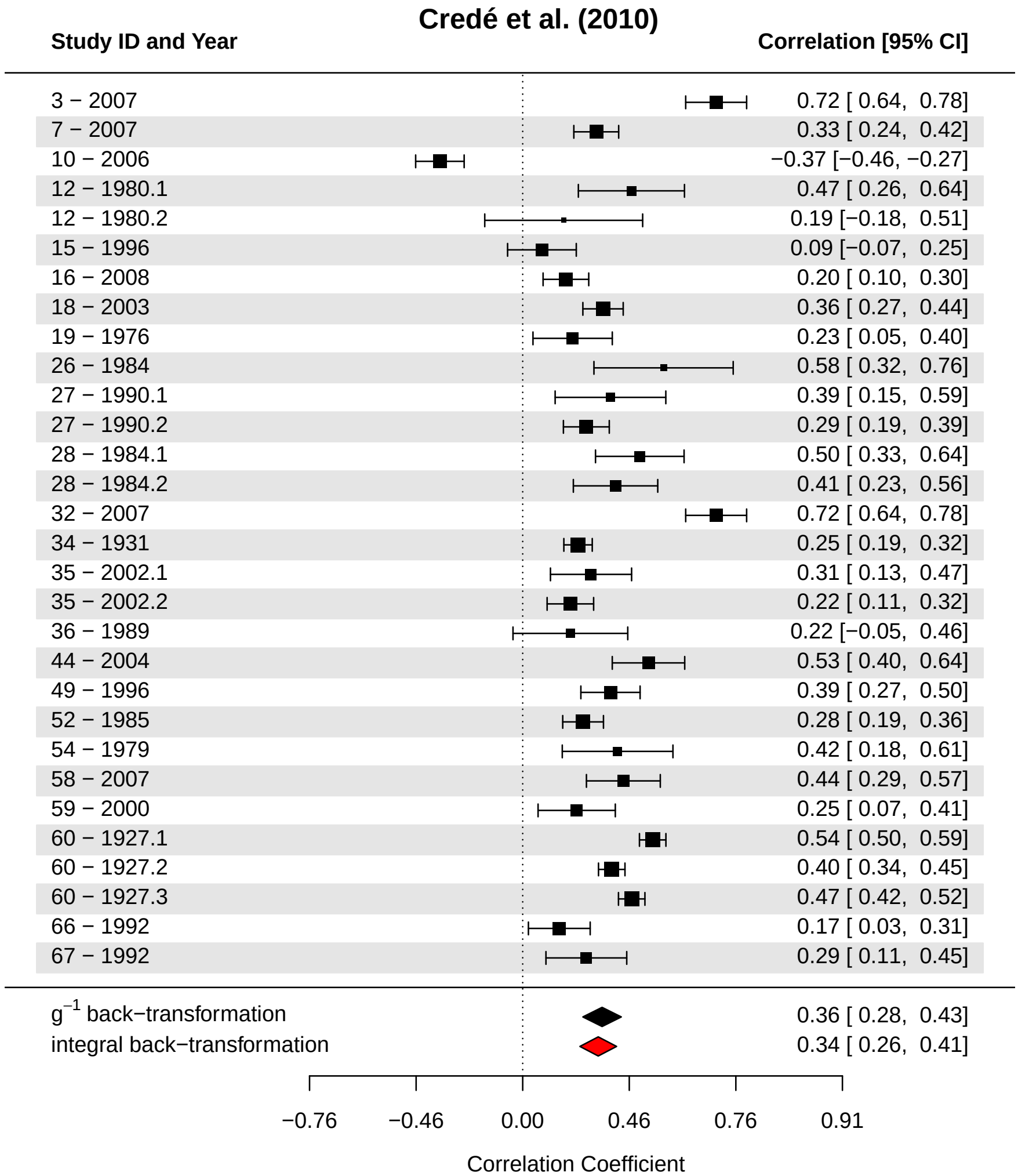


***Figure 10.*** *Forest plot of the Credé et al. meta-analysis*[3] *with* $g^{-1}$ *and integral back-transformation for Fisher's r-to-z transformation applied on correlations;* $\hat{\tau} = 0.24$.

## *B.4. Demir et al.*

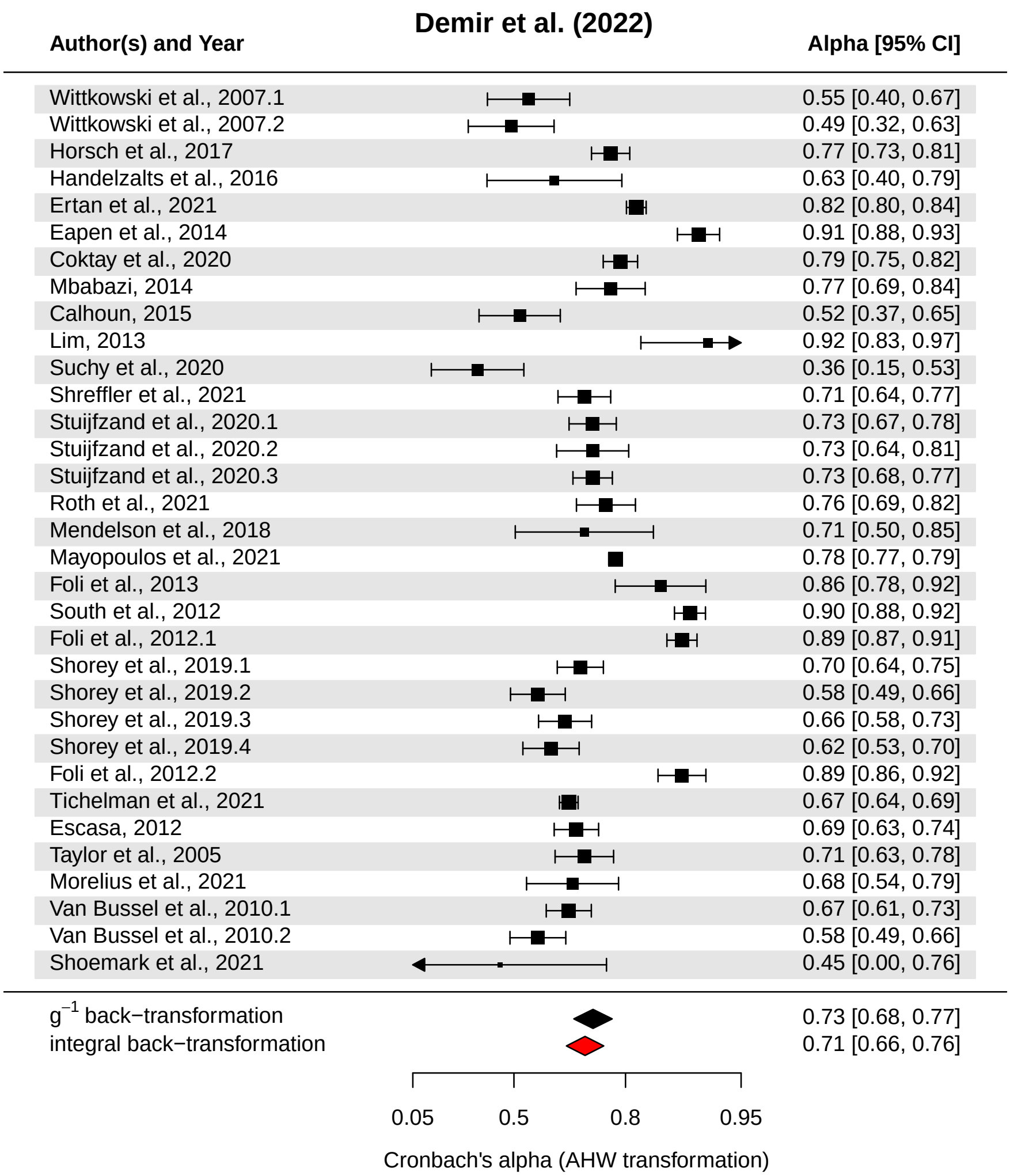


***Figure 11.*** *Forest plot of the Demir et al. meta-analysis*[4] *with* $g^{-1}$ *and integral back-transformation for the AHW transformation applied on alpha coefficients;* $\hat{\tau} = 0.01$.

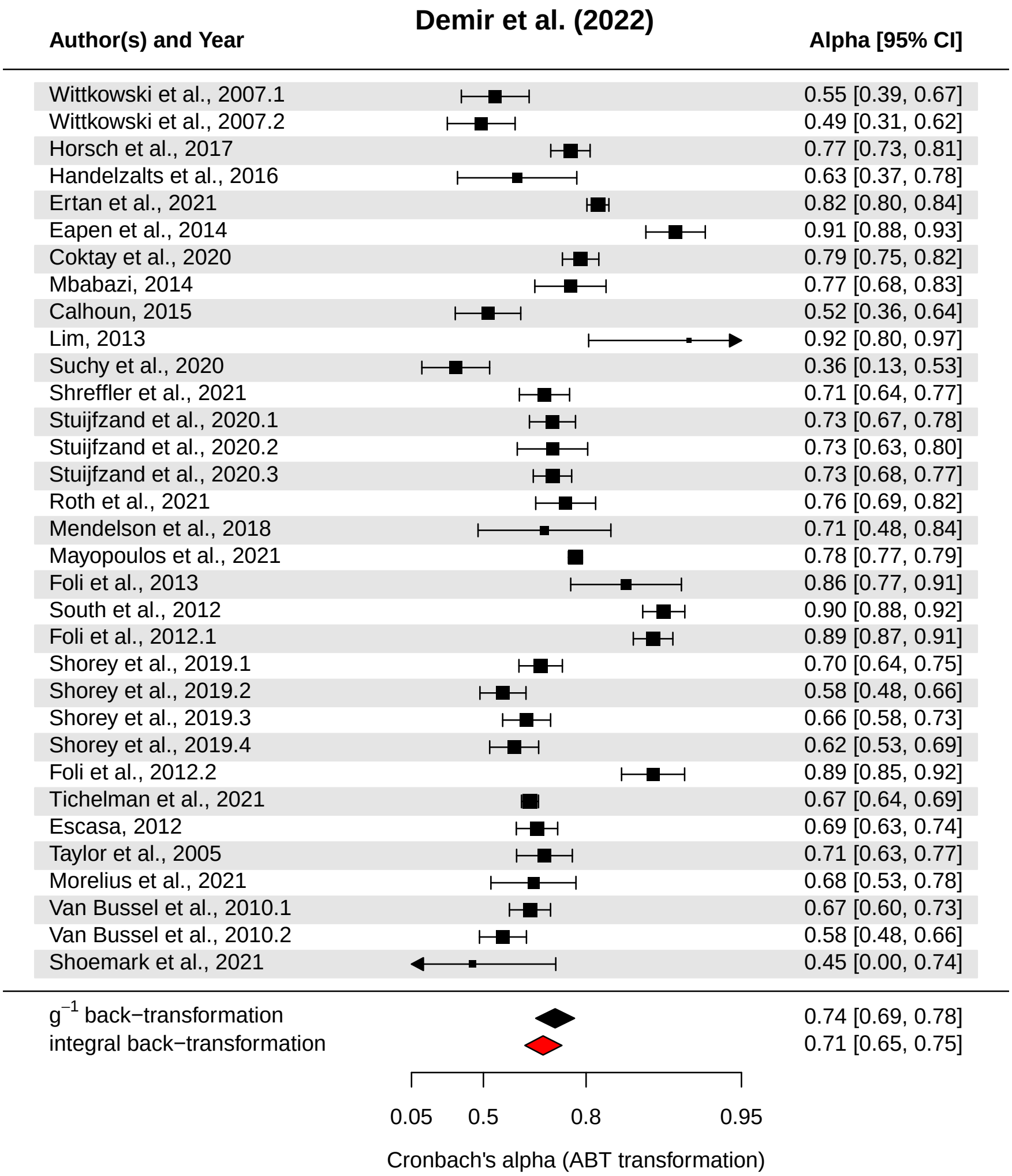


***Figure 12.*** *Forest plot of the Demir et al. meta-analysis*[4] *with* $g^{-1}$ *and integral back-transformation for the ABT transformation applied on alpha coefficients;* $\hat{\tau} = 0.48$.

## References

1. de Bruin M, Viechtbauer W, Hospers HJ, Schaalma HP, and Kok G. Standard Care Quality Determines Treatment Outcomes in Control Groups of HAART-adherence Intervention Studies: Implications for the Interpretation and Comparison of Intervention Effects. Health Psychology 2009;28:668–74.

2. Pritz MB, Zhou XH, and Brizendine EJ. Hyperdynamic Therapy for Cerebral Vasospasm: a Meta-Analysis of 14 Studies. Journal of Neurovascular Disease 1996;1:6–8.

3. Credé M, Roch SG, and Kieszczynka UM. Class Attendance in College: A Meta-Analytic Review of the Relationship of Class Attendance With Grades and Student Characteristics. Review of Educational Research 2010;80:272–95.

4. Demir E, Öz S, Aral N, and Gürsoy F. A Reliability Generalization Meta-Analysis of the Mother-To-Infant Bonding Scale. Psychological Reports 2024;127:447–64.
